# Reduced exacerbation frequency and prednisone dose in patients with ABPA and asthma treated with dupilumab

**DOI:** 10.1002/clt2.12081

**Published:** 2021-12-03

**Authors:** Tjeerd van der Veer, Marloes A. Dallinga, Johanna P. M. van der Valk, Jasper H. Kappen, Johannes C. C. M. in ’t Veen, Menno M. van der Eerden, Gert‐Jan Braunstahl

**Affiliations:** ^1^ Department of Pulmonary Medicine Franciscus Gasthuis & Vlietland Rotterdam The Netherlands; ^2^ Department of Pulmonary Medicine Erasmus MC Rotterdam The Netherlands; ^3^ Allergy and Clinical Immunology, Immunomodulation and Tolerance Group Inflammation Repair and Development Imperial College National Heart and Lung Institute London UK

**Keywords:** ABPA, allergic bronchopulmonary aspergillosis, asthma, dupilumab, ABPA, allergische bronchopulmonale aspergillose, asthma, dupilumab, ABPA, asma, Aspergilosis broncopulmonar alérgica, dupilumab

1

To the Editor,Allergic bronchopulmonary aspergillosis (ABPA) is a difficult‐to‐treat disease caused by airway inflammation triggered by an excessive allergic response to inhaled fungal spores. ABPA typically occurs in patients with asthma or cystic fibrosis and is characterized by dyspnea, cough, mucus plugs, and frequent exacerbations and is associated with bronchiectasis. High total serum IgE levels and high eosinophil counts are key biomarkers of ABPA.

To date, the cornerstone of ABPA treatment is systemic steroids. Azole antifungals are often used as an adjunctive treatment for nonresponders or as a steroid‐sparing agent. As steroids and azole antifungals often carry harmful side effects, it is of great importance to find alternative therapies.^1^

Here, we present a series of eight patients with a diagnosis of allergic ABPA and asthma who were treated with dupilumab, a novel biological therapeutic agent.

Dupilumab is an interleukin (IL)‐4 alpha receptor antagonist impeding IL‐4 and IL‐13 signaling, broadly inhibiting type 2 inflammation by counteracting IgE producing B‐cells and eosinophils.^2^ This gives dupilumab a rational mechanism of action in ABPA. Dupilumab efficacy in patients with ABPA and asthma has previously been reported in single case reports.^3^, ^4^ A post hoc analysis of the LIBERTY trial also showed reduced exacerbations in 18 participants treated with dupilumab who had serologic markers suggestive of ABPA but no formal diagnosis.^5^

Our retrospective case series approved by the institutional review board presents eight patients with allergic asthma as well as ABPA diagnosed by a pulmonologist according to the diagnostic criteria of the International Society for Human and Animal Mycology.^1^ All patients provided written informed consent for data collection.

Dupilumab prescription was indicated in these patients to treat underlying uncontrolled allergic eosinophilic asthma. The patients' number of exacerbations, maintenance prednisone dose, FEV1, total IgE levels, and eosinophil counts were compared between baseline and 6 months prior to first administration of dupilumab versus 6 months after start of dupilumab treatment. An exacerbation was defined as a worsening of symptoms for which a temporarily increased dose of steroids was prescribed.

Seven males and one female were included with a median age of 72 years. All patients had the diagnosis of ABPA for more than 1 year and were on maintenance prednisone treatment at baseline, except one patient. All patients used inhaled corticosteroids and bronchodilators. Seven patients had a history of azole antifungals treatment. All patients started dupilumab between March 2019 and July 2020, the maintenance dose was 300 mg every 2 weeks. The patients received between 4 and 21 months of dupilumab treatment.

Median FEV1 at baseline was 77% predicted (27%–96%), median total IgE was 1920 kU/L (66–4147 kU/L) and median baseline serum eosinophil counts were 0.4 × 10^9^ per litre.

After 6 months of dupilumab treatment, the number of exacerbations was significantly lower with a mean of 0.38 (SD 1.07) versus 2.5 (SD 0.52) during the six months before dupilumab treatment (paired samples *t*‐test, *t* (7) = 6.07, *p* < 0.001). After six months of dupilumab treatment, maintenance prednisone was discontinued in four patients and reduced in three other patients. Daily maintenance prednisone dose was compared for seven patients with mean dose at baseline of 7.9 mg/day (SD 3.66) versus 1.8 mg/day (SD 2.38) after six months of dupilumab treatment (paired samples *t*‐test, *t* (6) = 4.25, *p* < 0.01). The prednisone maintenance dose data of one patient could not be compared because of an unstable maintenance dose at baseline due to frequent exacerbations. After 6 months, five patients had a ≥10% increase in predicted FEV1, whereas the predicted FEV1 was unchanged in two patients and unavailable in one patient because of missed measurements due to the COVID‐19 pandemic. Five patients had a strong reduction in total serum IgE, while follow‐up measurements of serum IgE were not available for the other three patients. Eosinophil counts were unavailable for comparison in most patients (see Table 1).

**TABLE 1:** Patients characteristics and parameters at baseline, before and after starting dupilumab treatment

Parameter	Number of exacerbations		Prednisone maintenance dose (mg/day)		FEV1 %pred		Total IgE kU/L		Serum eosinophils (absolute × 10^9^/L)	
Patient sex, age	During 6 months before start dupilumab	During 6 months after start dupilumab	Base line	After 6 months	Base line	After 6 months	Base line	After 6 months	Base line	After 6 months
1. M, 83	3	0	10	0	84	103	1841	913	0.04	0.27
2. M, 78	1	0	10	2.5	92	94	4147	651	N/A	0.55
3. M, 52	3	0	10	0	95	109	994	474	0.21	0.11
4. M, 61	4	1	No stable dose	0	27	26	1046	531	0.55	N/A
5. M, 74	3	0	7.5	0	96	106	2324	N/A	1.84	N/A
6. F, 78	2	1	10	5	68	N/A	>2000	N/A	0.4	N/A
7. M, 69	1	0	7.5	5	69	88	66	N/A	0.00	0.07
8. M, 21	3	1	0	0	55	65	4598	1143	1.6	0.31

This is the largest reported case series of dupilumab treatment for diagnosed ABPA patients with a comparison of key outcome measures. Our findings indicate that dupilumab had strong clinical and biochemical effects. Most importantly, a significant reduction in the number of exacerbations along with a significant reduction in maintenance prednisone dose was observed in this case series. Side‐effects were limited, which is consistent with other studies which showed a favorable dupilumab safety and tolerability profile.^6^, ^7^

These results are limited by retrospective methods and the small number of patients. Results from a prospective phase III trial of dupilumab treatment for ABPA are not expected until the end of 2023 (Clinicaltrials.gov no. NCT04442269). However, based on this case series and strong immunological arguments, dupilumab seems to have strong beneficial clinical effects on the number of exacerbations and prednisone maintenance dose in asthmatic patients with a diagnosis of ABPA.

## CONFLICT OF INTEREST

Tjeerd van der Veer, Marloes A. Dallinga, and Johanna P.M. van der Valk declare no conflict of interests.
